# Adsorption Kinetics of Methyl Orange from Model Polluted Water onto N-Doped Activated Carbons Prepared from N-Containing Polymers

**DOI:** 10.3390/polym15091983

**Published:** 2023-04-22

**Authors:** Abdel-Basit Al-Odayni, Faisal S. Alsubaie, Naaser A. Y. Abdu, Haifa Masfeer Al-Kahtani, Waseem Sharaf Saeed

**Affiliations:** 1 Engineer Abdullah Bugshan Research Chair for Dental and Oral Rehabilitation, College of Dentistry, King Saud University, Riyadh 11545, Saudi Arabia; 2 Department of Chemistry, College of Science, King Saud University, Riyadh 11451, Saudi Arabia439106262@student.ksu.edu.sa (N.A.Y.A.);

**Keywords:** polypyrrole, polyaniline, nitrogen-containing polymers, activated carbon, adsorption kinetic, water treatment, methyl orange

## Abstract

This study aimed to assess the role of polymeric sources (polypyrrole, polyaniline, and their copolymer) of nitrogen (N)-doped activated carbons (indexed as PAnAC, PPyAC, and PnyAC, respectively) on their adsorption efficiency to remove methyl orange (MO) as a model cationic dye. The adsorbents were characterized using FTIR, SEM, TGA, elemental analysis, and surface area. The kinetic experiments were performed in batches at different MO concentrations (*C*_0_) and adsorbent dosages. The adsorption kinetic profiles of pseudo-first-order, pseudo-second-order (PSO), Elovich, intraparticle diffusion, and liquid film diffusion models were compared. The results showed a better fit to the PSO model, suggesting a chemisorption process. The adsorption capacity (*q*_e_, mg/g) was found to have increased as MO *C*_0_ increased, yet decreased as the adsorbent quantity increased. At the adsorption operating condition, including MO *C*_0_ (200 ppm) and adsorbent dose (40 mg), the calculated *q*_e_ values were in the order of PAnAC (405 mg/g) > PPyAC (204 mg/g) > PnyAC (182 mg/g). This trend proved the carbon precursor’s importance in the final properties of the intended carbons; elemental analysis confirmed that the more nitrogen atoms are in the activated carbon, the greater the number of active sites in the adsorbent for accommodating adsorbates. The diffusion mechanism also assumed a rate-limiting step controlled by the film and intraparticle diffusion. Therefore, such an efficient performance may support the target route’s usefulness in converting nitrogenous-species waste into valuable materials.

## 1. Introduction

Water pollution is a major environmental problem that brings countless challenges for the world and thus calls for immediate action. Pollutants can be chemicals, garbage, and microbes. Out of the various chemicals found in contaminated water, including organic substances, synthetic dyes are the most dangerous [1]. Dye pollution is a worldwide issue due to dyes’ hazardous nature. Further, color pollution caused by various dyestuffs is visible in the environment and is a source of visual pollution. Typically, industrial discharges are the primary source of colorants, which are usually a combination of chemical, physical, and biological pollutants. Of these, textile, papers, plastics, pharmaceutical, and food industries often produce significant amounts of dyes that are toxic to living organisms [2]. Dyes are marked as one of the most dangerous pollutants due to their potential carcinogenic and mutagenic impacts. They are highly soluble in water and hardly degrade under natural conditions, and thus endure for a long-time in the environment. Further, dyes aggressively influence the ecosystem by changing the water’s chemistry, decreasing sunlight penetration into water, driving undesirable effects on aquatic creatures, and drastically altering their biosystems [3].

Azo dyes are the most important chemical class of dyes, representing 70% of the world’s organic colorants and serving in diverse applications, including the food, textile, paper, and printing industries. They are relatively easy to synthesize, having nitrogen as the azo group, and their threat arises from their solubility in water. Methyl orange (MO) is a well-known sulfonated azo dye and is one of the most industrially used colorants. Furthermore, it is a laboratory pH indicator with a red-to-yellow color change range of 3.1–4.4 [4].

Various technologies have been employed to remove dyes from polluted water, including catalytic photodegradation, ozonation, coagulation, ion exchange, filtration, and adsorption [5,6,7]. Adsorption is the most competitive and applicable technique for dyestuff removal due to its ease of use, low cost, and high efficiency [5,7,8]. Thus, various adsorbents have been explored; however, each candidate adsorbent has its advantages and limitations, and no specific adsorbent has satisfied all the required conditions for efficient purification. In addition, favored adsorbents typically feature high surface areas, low toxicity, renewability, less waste by-products, low costs, and improved stability [5,9,10,11]. Activated carbon (AC) is one of the most researched adsorbents for water remediation and is featured with high porosity, tremendous capacity, is cheap, and, most importantly, is a reachable material [12,13]. It can be obtained by chemical or physical activation of various precursors, including natural and synthetic wastes.

Nitrogen (N)-containing polymers such as acrylonitrile-based thermoplastic (e.g., styrene-acrylonitrile, acrylonitrile butadiene-styrene), carriers such as polydopamine, conducting polymers (i.e., polyaniline (PAn), polypyrrole (PPy)), nylons (e.g., polyamides), etc., represent examples of N-rich precursors [12,13,14,15] from, e.g., wastes that can be converted into N-containing carbons. For instance, N-containing carbons based on conducting polymers have shown an exciting performance as adsorbents [13,16,17] for dye removal from wastewater. However, the end properties of activated carbons, including N-doped ones, depend on various factors such as the carbon source, the activator and the activation process [18]. Notably, activation using inorganic bases such as potassium hydroxide (KOH) has been reported to be efficient in producing effective adsorbents [4,17]. 

To our knowledge, no work has been conducted to analyze the role of N-containing precursors (i.e., PAn, PPy, and their copolymer) on the adsorption efficiency of the corresponding activated carbons. Therefore, this study aimed to synthesize PPy-based, PAn-based, and their copolymer-based KOH-activated carbons to assess the polymeric source role in the adsorbent efficiency for methyl orange removal in terms of kinetic processes at various conditions. The performance was studied through multiple kinetic models and the associated mechanism was discussed based on their physicochemical and structural properties.

## 2. Materials and Methods

### 2.1. Materials

The monomers pyrrole C_4_H_5_N (Py, +98%) and aniline C_6_H_7_N (Ani, 99%), the initiator ammonium persulfate (NH_4_)_2_S_2_O_8_ (APS, 98%), and carbon activator potassium hydroxide (KOH, 85%) were purchased from Alfa Aesar (Karlsruhe, Germany). The solvents absolute ethanol (99.5%) and hydrochloric acid (HCl, ~36%) were procured from Fisher Chemical (Loughborough, UK). The sodium salt of methyl orange C_14_H_14_N_3_NaO_3_S (MO, 99.8%) was obtained from BDH Chemicals Ltd. (Poole, UK). All chemicals were used as received without additional treatment unless stated otherwise. Distilled water was used wherever required.

### 2.2. Preparation of Adsorbents

The PAn, PPy, and poly(An-co-Py)-based activated carbons (denoted as PAnAC, PPyAC, and PnyAC) were synthesized using a method reported in the literature [7]. Briefly, 0.08 mols of the monomeric precursor were charged into 1 L of precooled (5 °C, ice bath) 0.1 M HNO_3_ and stirred for 30 min. Then, 30 mL of a freshly prepared cold aqueous solution of the oxidant APS (0.12 mol) was added drop-by-drop. Although the polymerization product was immediately observed at room temperature, it was left overnight for completeness, then filtered and adequately washed with ethanol and water. The synthesized polymers were mixed with 4-times KOH and carbonized using a horizontal MTF Carbolite 12/38/250 (Walf Laboratories, Hope, UK) under a nitrogen atmosphere, heating speed of 3 °C/min, and activation time and temperature of 2 h and 650 °C. The obtained nitrogen-doped graphene oxides were sufficiently washed using 0.5 M HCl and distilled water until neutrality, then dried at 100 °C for 24 h.

### 2.3. Assessment of Surface Charge

The surface charge of prepared adsorbents was assessed using a pH-drift technique described in the literature [19]. In a typical method, a buffered solution of 0.1 M NaNO_3_ was modified to pH values between 2 and 10 using 0.1 M HCl or NaOH (pH_i_). Then, 15 mL of the prepared pH_i_ solutions were mixed with 15 mg of the target adsorbent and intermittently agitated for 24 h at room temperature. The adsorbents were filtered, and the final pHs were determined (pH_f_). Thus, the pH at which the surface is uncharged (pH_PZC_) was determined by plotting pH_i_ vs. pH_f_. 

### 2.4. Adsorbate Solution

A stock solution of 1000 ppm MO was prepared in distilled water. The experimental solutions were obtained by dilutions, and their pHs were recorded. For colorimetric measurement afterward, the λ_max_ of MO was determined from a scanned full electronic spectrum (200–800 nm) against a blank sample of distilled water and found to be at 465 nm. Based on Beer–Lambert law, a standard curve was established for MO concentrations of 5, 10, 15, 20, and 25 ppm (*R*^2^ = 0.9815) and used for computing the MO concentration at various stages.

### 2.5. Adsorption Kinetic Experiments

The adsorption kinetics of MO (*C*_0_ = 50, 100, and 200 ppm) onto the investigated adsorbents (PAnAC, PPyAC, and PnyAC) of various doses (20, 40, and 80 mg) were evaluated over the time course of 0–180 min at a constant shaking speed of 150 rpm, a processing temperature of 24 ± 2 °C, and a solution pH of 6.2–6.6. The effect of MO *C*_0_ on the adsorption kinetics was studied using the 40 mg adsorbent dose, while the dosage screening was performed for the 100 ppm *C*_0_, keeping other parameters constants. The experiments were carried out in batches; thus, 250 mL of MO dye was agitated and during which 3 mL aliquot was taken out for concentration measurement at predefined intervals, then centrifuged to separate adsorbent particles, measured for MO concentration, re-suspended, and returned into the adsorption solution. 

### 2.6. Characterization

The adsorbent structural and surface characteristics were studied using various techniques. The Fourier transform infrared (FTIR) spectra were obtained on a Nicolet iS10 from Thermo Scientific (Madison, WI, USA) using attenuated total reflection (ATR) mode (diamond) over the frequency range of 4000–500 cm^−1^ at 4 cm^−1^ and 16 cycles per spectrum. The BET (Brunauer–Emmett–Teller)-specific surface area was measured on a Gemini VII2390 V1.03 machine (Micromeritics, Norcross, GA, USA), operated in single-point and multi-point modes; before each BET measurement, the sample was degassed at 150 °C for 3 h. The surface morphology was imaged using a scanning electron micrograph (SEM) (JSM-6360 LV, JEOL, Tokyo, Japan). Thermal analysis was carried out using TGA/DSC 1 Star thermogravimetric analysis (TGA) from Mettler Toledo (Columbus, OH, USA); the sample was heated from 25 to 800 °C at a heating rate of 10 °C/min under a N_2_ atmosphere of 20 mL/min. The pH values were recorded on a benchtop Orion 3 Star pH meter from Thermo Scientific (Beverly, MA, USA), which was calibrated before measurement using two supplied buffers 4 and 7. The dye concentration was measured using a Hitachi U-2910 double-beam ultraviolet/visible (UV/Vis) spectrophotometer (Tokyo, Japan).

### 2.7. Adsorption Kinetic Models

The data obtained from kinetic experiments for various amounts of MO (adsorbate) and AC (adsorbent) were subjected to theoretical modeling. The linear and nonlinear (Table S1) equations of pseudo-first-order (PFO), pseudo-second-order (PSO), Elovich, intra-particle diffusion (IPD), and liquid film diffusion (LFD) models were selected to fit the experimental data. The magnitude of correlation coefficient (*R*^2^) was used for goodness fitting assessment; when *R*^2^ approaches the unity, the predicted values become closer to the experimental ones. Hence, the corresponding model is most applicable for describing the adsorption system.

#### 2.7.1. Pseudo-First-Order Kinetic Model (PFO) 

PFO, also called the Lagergren model, is adapted to describe the adsorption mechanism related to physisorption and diffusion [20]. The model assumes one adsorption site for each adsorbate molecule [21], and the adsorption with time is directly proportional to the instant capacity [22,23]. The corresponding nonlinear equations are given in Table S1. The corresponding parameters can be obtained by plotting the logarithmic values of the remained capacity (ln(*q*_e_ − *q*_t_)) with time [24].

#### 2.7.2. Pseudo-Second-Order Kinetic Model (PSO)

The PSO model assumes that one molecule is sorbed onto two active sites of the adsorbent [25] and the adsorption is controlled by the chemisorption mechanism, which involves valency forces through electron sharing or transfer between the adsorbent and the adsorbate [26]. Additionally, the reaction rate commonly depends on the adsorbed amount [27]. As seen in Table S1, the PSO parameters can be obtained by plotting the experimental *q*_t_ or *t*/*q*_t_ against *t*. The rate constant (*k*_2_) and the initial rate (*h*) can be calculated from the PSO equation.

#### 2.7.3. Elovich Kinetic Model

The Elovich equation (Table S1) is satisfied in the chemical adsorption process and is suitable for systems with energetically heterogeneous surfaces [28]. When the surface coverage is low, the effect between adsorbed species does not substantially affect the kinetics [29]. Additionally, the sorption energy raises linearly with the surface coverage according to the Arrhenius equation [30]. The initial rate and the extent of adsorption are described by parameters 𝛼 and 𝛽, respectively, and 𝛽 can be used to assess the chemisorption process as well. 

#### 2.7.4. Intra-Particle Diffusion Kinetic Model (IPD)

Kinetic data were further analyzed using the IPD model given by Webber and Morris [31]. It is an empirical model that describes the diffusion mechanism and adsorbate transfer through the internal pores. As shown in Table S1, the adsorbate uptake is proportional to the root of time (*t*^0.5^) rather than the contact time, t. By plotting *q_t_* vs. *t^0.5^*, the initial rate constant (*k*_id_) and the y-intercept (C), or the thickness of the boundary layer, can be obtained. These parameters are essential for identifying the adsorption mechanism and predicting the rate-controlling step. If the IPD is the limiting step, the plot will be linear and pass through the origin, and if not, then some other mechanism will be involved. 

#### 2.7.5. Liquid Film Diffusion Kinetic Model (LFD)

The rate-limiting step is an important factor for proper corroboration of the adsorption mechanism. The adsorption process is governed by a solute transfer process represented by external mass transfer, intraparticle diffusion, or both. Hence, the liquid film diffusion model, the Boyed kinetic expression [32], was used. To quantify the magnitude of mass transfer, the term containing the fractional capacity (−ln(1 − F)) was plotted vs. *t*, and from which the film diffusion rate constant (*k*_fd_, min^−1^) and boundary layer (*C*_fd_, mg/g) were calculated. 

## 3. Results and Discussion

### 3.1. Adsorbents Properties

#### 3.1.1. Physicochemical Properties

**Figure 1:**
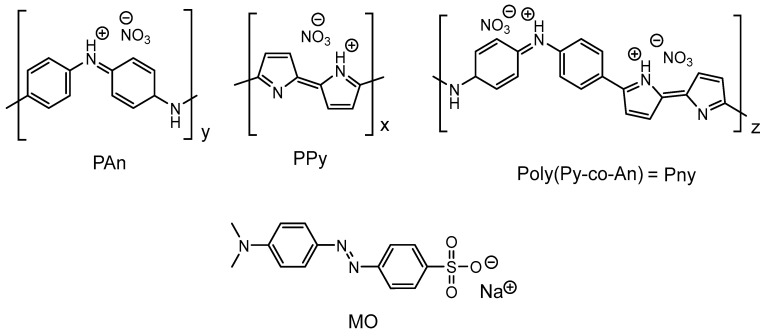
Chemical structure of PPy, PAn, PnyAC, and MO.

The chemical structure of the synthesized polymers (PAn, PPy, and poly(An-co-Py)) and their nitrogen-doped graphene oxides counterparts (adsorbents: PAnAC, PPyAC, and PnyAC) are shown in Figure 1, along with the dye under investigation (MO). Generally, the molecular structures of the polymers and the doped (protonated) ones proposed in the literature varies to a certain degree. However, there is agreement that both reduced and oxidized constitutional units are present after deprotonation while the delocalization of charges is still open to discussion. Figure 1 is a simplified presentation of the doped structures, showing the protonated chains and the NO_3_^−^ ion counterparts (polymeric salts) [33]. The chemical structure of the MO dye is also depicted in Figure 1 as a salty azo-structure which can form its anionic form upon dissolution at a neutral pH.

The result of the elemental analysis is presented in Table 1. Evidently, the ACs’ structure has retained a high content of heteroatoms N and O [4,17,34], providing the product with some essential functional groups representing part of the adsorbent active sites. Moreover, the PAnAC adsorbent has the highest O and N content, followed by the copolymer-based one (PnyAC), suggesting high active sites and potentially explaining the obtained high adsorption capacity of PAnAC. The possible explanation for the variation in elemental composition could be the oxidative simplicity differences between the polymeric precursors, which is somehow related to the folding type; otherwise, it might be a result of the insufficient mixing of KOH with the polymer prior to carbonization. 

The specific surface area, as well as the capacity toward the uptake of carbon dioxide (CO_2_), were also measured and found to be in the order of PAnAC > PPyAC > PnyAC (Table 1), in consistence with their adsorption capacity for MO dye as discussed below. 

**Figure 2:**
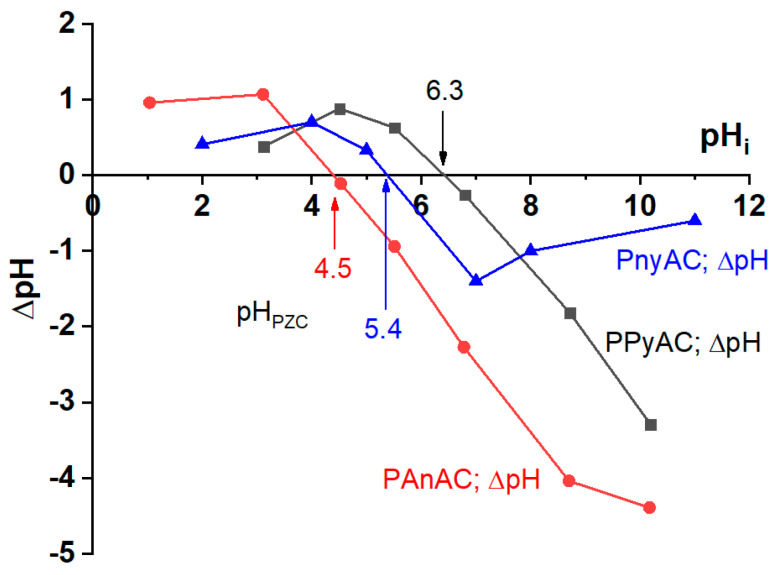
pH at point of zero charges for PAnAC [17], PPyAC, and PnyAC adsorbents.

The pH_PZC_ of the three adsorbents was measured by the batch equilibrium method [16], as shown in Figure 2. It was estimated at pHs of 4.5, 5.4, and 6.3 for PAnAC, PPyAC, and PnyAC, respectively. Typically, the surface is neutral at pH_PZC_, below which the surface charge is positive but negatively charged above it. Noticeably, the pH_PZC_ of PnyAC is in-between those for PAnAC and PPyAC; however, the capacity for MO adsorption was not straightforward with pH_PZC_, a case that could reflect some differences in the macromolecular structure of the adsorbents due to dissimilar precursors. In addition, the kinetic-based capacity of the adsorbents was measured at an unadjusted pH of 6.4 ± 2 (measured before the addition of the adsorbents), which may support higher adsorption onto PAnAC than others; however, this case was not supported by the observed capacities of PPyAC and PnyAC and other factors, such as chemical and three-dimensional structures, which could be the driving force of each adsorbent performance. The pH value for the MO dye-contaminated water solution was about 6.4, which is close to or slightly higher than the pH_PZC_.

#### 3.1.2. Surface Morphology

**Figure 3:**
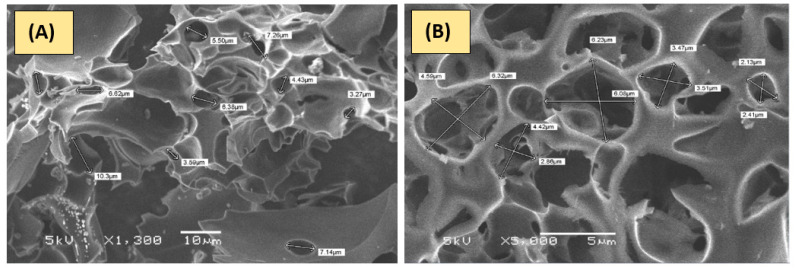
SEM micrograph of (**A**) PPyAC and (**B**) PnyAC.

The surface texture was observed with SEM at an accelerating voltage of 5 kV, as shown in Figure 3. The micrographs of the as-synthesized PAnAC [17], PPyAC, and PnyAC have revealed a rough surface with peel-like structures. Furthermore, the surface combined with pores of relatively large and irregular shape and with an average diameter of about 3–5 μm. According to the previous analysis [35], the resulting activated carbons are of nitrogen-doped graphene sheets type with surfaces that are seemingly similarly rugged [7].

#### 3.1.3. FTIR Spectra

**Figure 4:**
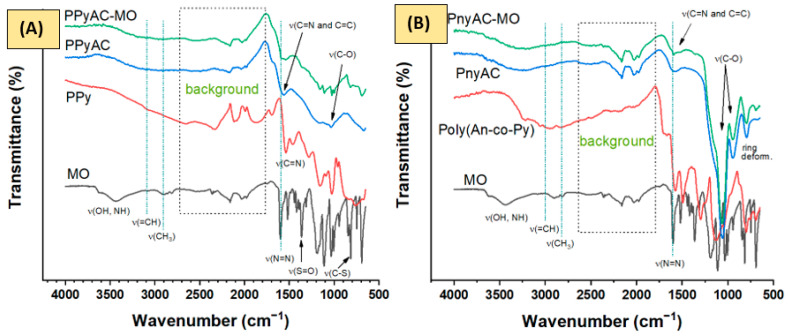
FTIR spectra for methyl orange (MO), polypyrrole (PPy), poly(An-co-Py), polymer-based activated carbons (PPyAC and PnyAC), and AC-loaded MO (PPyAC-MO, and PnyAC-MO). (**A**) gathers the FTIR spectra of PPy-based and (**B**) for poly(An-co-Py)-based parts.

Figure 4 shows the spectra of the investigated polymers (PPy and poly(An-co-Py), polymer-based activated carbons before (PPyAC and PnyAC) and after (PPyAC-MO and PnyAC-MO) the adsorption of MO and MO dye. PAn-based spectra have been reported in Al-Odayni et al. [17]. The spectra of MO, PPy, PAn, PPyAC, PAnAC, PPyAC–MO and PAnAC–MO were consistent with the literature [4,17,36,37,38]. The characteristic peaks of MO dye were clear at 3626 and 3431 cm^−1^ for adsorbed water hydroxyl (OH), 3030 cm^−1^ (=CH), and 2900–2811 cm^−1^ (CH_3_) stretching modes and at 1519 cm^−1^ for C–H bending. The benzene ring of MO is affirmable via ring deformation at 1034, 1005, and 846 cm^−1^, while substitution is proven by the peak at 816 cm^−1^ [36]. Peaks corresponding to the azo group are seen at 1599 (-N=N-) and 1112 cm^−1^ (-C-N). The sulfonic nature is confirmed by peaks at 1363 and 692 cm^−1^ attributed to S=O and -C-S- stretching modes, respectively. As can be seen, PPy spectrum demonstrated peaks at 1538, 1457 cm^−1^ for C=N stretching, and at 1280, 1158, and 1027 cm^−1^ for C=C and C-N stretching and =C-H bending modes, respectively. The observed peaks at 762 and 674 cm^−1^ were assigned for aromatic ring deformation while the peaks at 2326 and 961 cm^−1^ were assigned for C=NH-C of the immonium group in the doped structure [39]. The PPyAC spectra revealed fewer peaks than its precursor due to conversion into activated carbon; however, the characteristic ones at 1688 (C=O stretching), 1557 (C=N and C=C stretching), 1161 (C-N stretching), 1033–870 and 667 cm^−1^ for C-H bending and aromatic skeletal bands, respectively, were readable. After adsorption, peaks for C=N (1557 cm^−1^) and C-O (1035 cm^−1^) were shifted to 1539 and 1157 cm^−1^, respectively, supporting their involvement in the adsorption mechanism. New peaks in the fingerprint region were also observed and attributed to the adsorbed MO [4].

The characteristic peaks of PAn [17] were reported at (cm^−1^) 3219 (NH stretching band) [40], 3048 (=CH stretching), 2917 (CH_3_ stretching), 2328 (C=NH-C, immonium), 1569 and 1484 (C=N and C=C stretching in, respectively, quinoid and benzenoid rings), 1289 and 1241 cm^−1^ (C-N-C and C-N stretching), 1036 and 876 cm^−1^ (C-H bending, its broadening may indicate the contribution of peaks from dopant NO^3−^), and 792 cm^−1^ (1,4-disubstituted phenyl ring [41]). The pattern of PAnAC and PAnAC-MO spectra were similar to that of PPyAC and PPyAC-MO, respectively, indicating alike or closer structures.

The spectrum of the co-polymer is almost similar to its homopolymers, with characteristic peaks observed at 1575, 1494, 1299, 1126, 1043, 799, 755, and 690 cm^−1^. However, the spectra of PnyAC and PnyAC-MO revealed poorer peaks than homopolymer-based ones. Nevertheless, peaks for OH (broad around 3300 cm^−1^), C=N and C=C stretching at 1595 cm^−1^, 1065, and 949 cm^−1^ for C-O stretching, and 798 and 692 cm^−1^ for ring deformation were observed. Evidently, as the adsorption of PnyAC is low, its spectra showed little or no visible difference from that after adsorption (PnyAC-MO).

#### 3.1.4. Thermal Analysis

**Figure 5:**
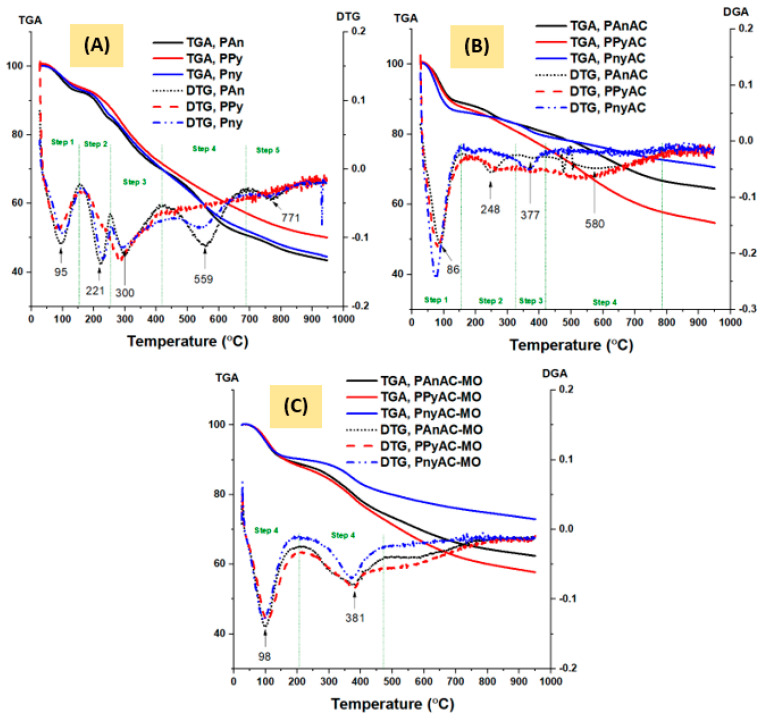
TGA and DTG curves of polymers (precursors) (**A**), activated carbons (adsorbents) (**B**), and methyl orange-loaded adsorbents (**C**).

Thermal stability and thermal decomposition profiles for the materials under investigation were analyzed under an inert atmosphere of nitrogen gas using the typical thermogravimetric analysis (TGA) method, as illustrated in Figure 5. The subfigures collect, besides the corresponding DTG curves, the thermograms of the polymers (PPy, PAn, and PnyAC), adsorbents (activated carbons; AC), and MO-loaded adsorbents. Furthermore, the predicted decomposition steps are summarized in Table S2. The decomposition curves can be exhibited in, e.g., five steps for polymers, four for ACs, and two for MO-loaded ACs. By comparison, the stability was found in the order PPy > Pny ≥ PAn, PnyAC > PAnAC > PPyAC, and PnyAC-MO > PAnAC-MO > PPyAC-MO depending on the DTG values of step 2. In the case of polymers, the decomposition trend can be assigned to the slight difference in the chemical structure and geometry which visibly support higher stability for PPy followed by copolymer. The higher stability for the copolymer-based AC suggests the absence of adequate susceptible groups, including nitrogen-based groups as shown in Table 1. This may elucidate why PnyAC showed the lowest adsorption capacity. Likewise, PAnAC elemental analysis indicated a higher nitrogen content than PPyAC; thus, its high capacity is in accordance with the high number of heteroatoms, specifically nitrogen atoms (Table 1). After adsorption, the trend of residual carbon content is still the same. However, the values after adsorption were higher, probably due to the higher content of volatiles adsorbed on ACs, as indicated by the mass percent loss in the first step.

### 3.2. Adsorption Studies

#### 3.2.1. Effect of Solution pH

**Figure 6:**
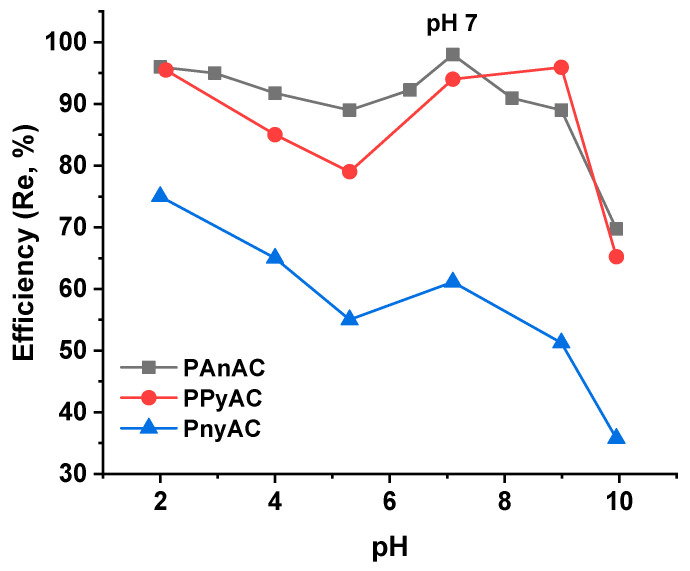
Solution pH effect on adsorption efficiency of the three adsorbents.

The pH effect of polluted water on the adsorption efficiency was also assessed. The experiments were carried out for MO dye *C*_0_ of 200 ppm, MO volume 25 mL, adsorbent dose 15 mg, agitation speed 150 rpm, agitation time 24 h, and at room temperature, while the pH value was varied from 2 to 10. As shown in Figure 6, the adsorption has two peaks at 2 and 7 pHs and a minimum of around 5. However, the kinetic study was performed at pHs close to 7 to avoid pH adjustment of pollutant solutions (~pH 6.4). The removal percentage (Re%) steadily decreased with pH increase from 2 to 5, then increased, almost showing a peak at pH 7, beyond which the efficiency was rapidly reduced. This behavior could be explained in terms of surface property alteration at different pH; for instance, the adsorbent is highly protonated at low pH; therefore, electrostatic attraction is expected to drive high adsorption of MO. By increasing the pH to the pH_PZC_, the adsorption decreased due to the depletion of positive sites. However, by further increasing the pH, the efficiency increased up to about pH 7, a case that may suggest a contribution of other mechanisms such as physical interaction rather than electrostatic ones. Otherwise, it is worth mentioning that the agglomeration event of reduced graphene oxide sheets commonly decreases in the natural and alkaline environment [42,43]. Even though such behavior is not always the case, the repulsion force between sheets may promote an exposure of more active sites, which could lead to the observed altered elevated efficiency over pH 5–7; hence, as the pH of unadjusted MO solutions was about 6.2–6.6 and the efficiency was close to the natural pH, the following experiments were carried out without pH adjustment. The drop in the adsorption efficiency beyond pH 7 is a result of electrostatic repulsion between the negatively charged surface and anionic dye molecules [44]. In all pHs, the efficiency was in the order of PAnAC > PPyAC > PnyAC, a trend that is consistent with the data obtained from the kinetic experiments detailed below.

#### 3.2.2. Effect of Adsorbate Initial Concentration

**Figure 7:**
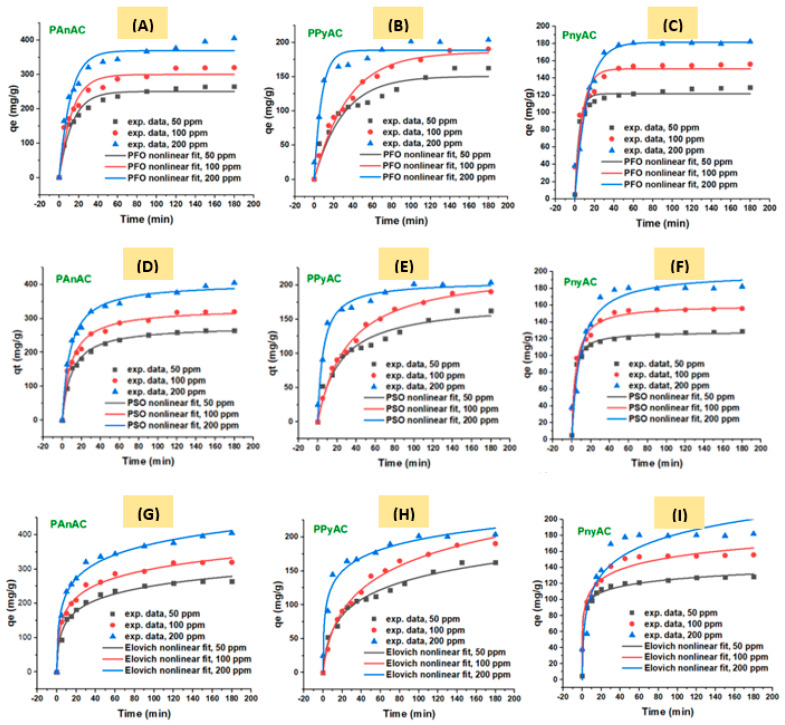
Kinetic adsorption plots of (**A**–**C**) pseudo-first-order (PFO), (**D**–**F**) pseudo-second-order (PSO), and (**G**–**I**) Elovich models.

**Figure 8:**
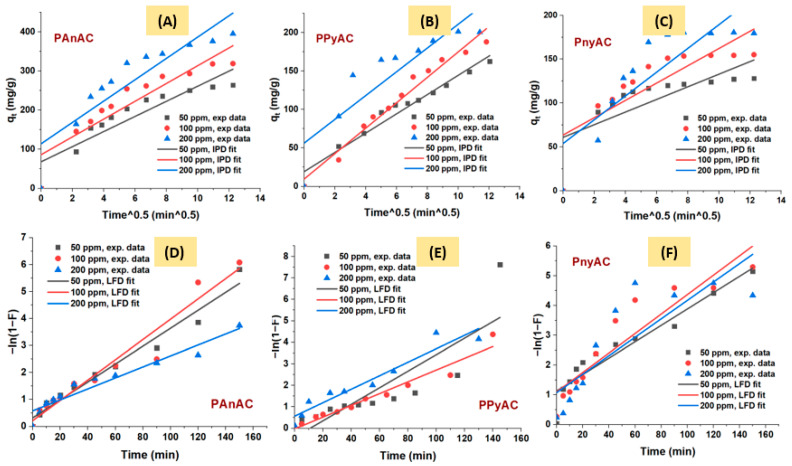
Kinetic plots of (**A**–**C**) intraparticle diffusion (IPD) and (**D**–**F**) liquid film diffusion (LFD) models.

The time-dependent adsorption profiles for the removal of MO dye by the targeted activated carbons (PAnAC, PPyAC, and PnyAC) were obtained at different initial dye concentrations (50–200 mg/L) combined with the following constants: 40 mg adsorbent dose, 250 mL MO volume, 150 rpm agitation speed, 24 ± 2 °C operating temperature, 6.2–6.6 solution pH, and 0–180 min contacting time. For adequate analysis, the collected data were modeled using PFO, PSO, Elovich, IPD, and LFD equations as shown in Figure 7 and Figure 8. Moreover, the linear models were also computed and the results were summarized in Figures S1–S3 and Table S3.

It is obvious that the adsorption was fast in the first few minutes (ca. 15 min) and then continued with a lesser rate by about 30 min, a period over which about two-thirds of adsorbent capacities were occupied with MO molecules. Such behavior is commonly associated with the charge interaction mechanism occurring on the adsorbent surface [4]. After that, adsorption was slowly developed until equilibrium (~60 min). It was found that the uptake rate was reduced when MO *C*_0_ increased from 50–200 ppm, and reaching equilibrium was delayed accordingly. However, the adsorption capacity was increased when MO *C*_0_ increased. For example, the obtained experimental capacity (*q*_e, exp_. (mg/g)) of PAnAC was raised from 264.7 to 405.0 (mg/g) as MO *C*_0_ increased from 50 to 200 ppm. The same trend was observed with PPyAC and PnyAC adsorbents. By comparison, PAnAC had the highest capacity followed by PPyAC and PnyAC. This observation can be correlated with the physical properties observed for each adsorbent, such as surface area and nitrogen content, and morphological properties such as shape and roughness. According to the literature [45,46], the SEM-based surface morphology of the copolymer poly(An-c-Py) is entirely different from that of the homopolymers (PAn and PPy) due to the effect of monomers molecules arrangement in the microstructures. Therein, authors have observed more aggregated particles for the copolymer, a case that may drive less surface area of the final carbonaceous materials and, thus, lower the capacity for CO_2_ and MO accommodation. In support of these findings, a previous report concerning their CO_2_ adsorption capacities has revealed similar trends for the three adsorbents [35]. Additionally, it was found that reaching equilibrium was quicker when the capacity was low.

According to the coefficient of determination (*R*²) given in Table 2, the data best fit the PSO kinetic model. Furthermore, the calculated PSO-based capacity (*q*_e, calc_) was closer to the experimental values (*q*_e, exp_) than PFO and Elovich, indicating that the adsorption mechanism is best described by the PSO model. Similar observations were found when the linear fit was performed, as given in Figures S1–S3 and Table S3. However, the linear models have shown poor *R*^2^ with either curtail (e.g., for PnyAC) or overestimation (e.g., for PAnAC and PPyAC) of the capacity than that from nonlinear equations. Additionally, in line with the idea that adsorption rate constants are inversely proportional to adsorbate initial concentrations, the k_2_ values were found to increase as MO increased. However, this was not always the case, and opposite results were reported [47].

Figure 8 represents IPD and LFD models for the MO adsorption onto the target adsorbents. The calculated parameters are summarized in Table 3. As can be seen, the two models do not pass through the origin, indicating that neither IPD nor LFD is the rate limiting step in the adsorption process. The high values of the IPD intercepts (C_id_, boundary layer) could suggest a higher contribution from LFD models than IPD. This argument can be supported by the R^2^ values, which were also higher for LFD models. 

#### 3.2.3. Effect of Adsorbent Dosage

**Figure 9:**
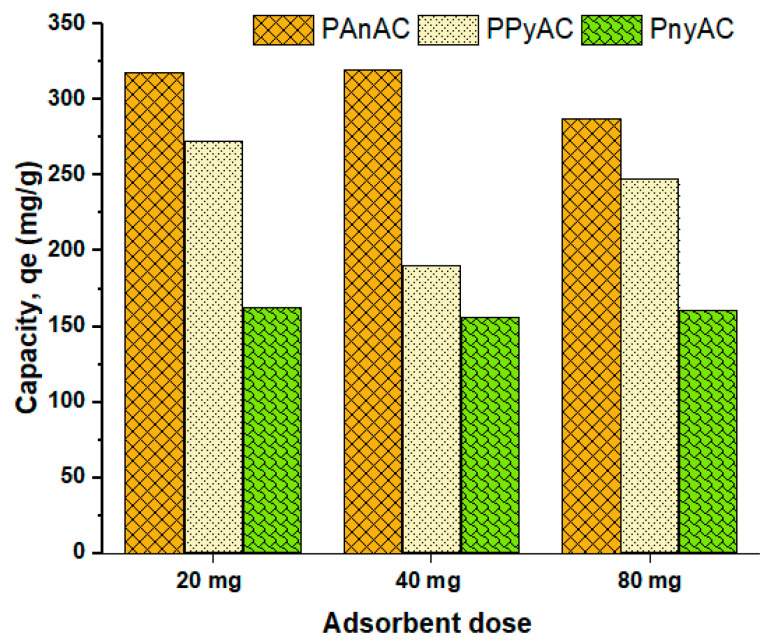
Histogram illustration of adsorption capacities of PAnAC, PPyAC, and PnyAC at dosages of 20, 40, and 80 mg per 250 mL MO volume.

The effect of the adsorbent dose (20, 40, and 80 mg) on the removal of MO dye was also investigated on a kinetic setup with the following conditions: a batch of 250 mL MO working volume, 100 ppm MO concentration, 150 rpm agitation speed, 24 ± 2 °C operating temperature, and 0–180 min contact time. The obtained results, in terms of adsorption capacities, are presented in Figure 9 and Table S4. As can be seen, the adsorption capacity for PAnAC (318.09, 319.73, and 287.73 mg/g) was the highest, followed by PPyAC (273.34, 190.30, and 247.76 mg/g) and PnyAC (163.04, 155.93, and 161.04 mg/g) for the tested doses of 20, 40, and 80 mg/250 mL MO, respectively. 

### 3.3. Suggested Adsorption Mechanism

To explore the potential of using an adsorbent, it is important to assess the kinetics of the adsorption. It is a time-dependent process that typically involves three stages: (i) a liquid film diffusion phase during which the sorbate molecules transfer through the boundary layer to the sorbent exterior surface; (ii) an intraparticle diffusion stage, which involves the transport of adsorbate particles from the outer surface to internal pores; and (iii) a sorption process, which is known to be fast and cannot be considered as a rate-limiting step. As discussed above, the adsorption kinetic is dominantly PSO type, which supposes multilayer coverage and a chemisorption process. The initial rate of adsorption was fast, as given by the PSO and Elovich constants *h* and *α*, and increased as *C*_0_ increased. The result of diffusion models indicated that neither IPD nor LFD is the sole rate-limiting step. Furthermore, the two-stage plots of IPD depicted in Figure S4 and Table S5 also supported poor contribution of both liquid film and intraparticle diffusions into the rate limiting step [17,48]. Furthermore, according to the goodness of fit, which was assessed via the values of the coefficient of determination (*R*^2^), the LFD was better at describing the experimental data than IPD, suggesting a higher contribution into the kinetic operating condition. Hence, some other mechanism, such as migration from the bulk and adsorbate–adsorbent interaction, may effectively participate into the adsorption process. It is known that the migration of the particles from the bulk of the solution to the external surface of the adsorbent can be reduced by improving agitation speed, while the interaction stage cannot be treated as the lowest step. Generally, the adsorption mechanism is more complicated than simplified above, and many factors may be implicated such as the adsorbate structure and functional groups, surface morphology of the adsorbents, and the nature of the interaction process [49]. Chemically, MO is a solid dye, crystallized in salt form, having an azo-structure that plays as an anionic indicator in solution. It is supposed that, while the pH is on the acidic side, the conditions support better adsorption performance with one peak close to neutrality; however, the experimental surface zero charge was slightly below pH 7. Thus, an additional mechanism other than electrostatics may also promote the adsorption, such as the physical and mechanical ones. Above pH 7, the adsorption declined due to repulsion with the negative sites on the adsorbent. The effective functional groups on the adsorbents are described in Section 3.1.3. The spectra of the coursed activated carbons have shown the presence of C=O, OH, NH, and N=C, which are effectively involved in electrostatic interactions and thus cannot be ignored.

### 3.4. Relative Performance of the Prepared Adsorbents

To evaluate the performance of the as-prepared nitrogen-rich activated carbons, their adsorption capacities were compared with a list of adsorbents reported in the literature, of which the following were taken into consideration: the structural similarity of the selected adsorbents, the closer operating condition of the adsorption process, and kinetic-based capacity values, as shown in Table 4. As can be seen, the N-doped activated carbon [50] have a higher capacity for MO compared to non-doped carbon at similar conditions. Furthermore, the synthesized ones performed better in the removal of azo dyes such as MO and reactive violet 5 [50,51,52]. The capacities of the investigated polymeric-based activated carbons are well-positioned in the list [53,54,55], showing comparable performance to the most reported adsorbent in Table 4. This confirms that nitrogenous adsorbents are more active, holding higher functional groups and active sites for MO settling. Adsorption of MO onto the N-containing polymers, i.e., PAn and PPy, was also compared. As can be seen, the adsorption capacities of PAn [56] and PPy [57] were 111 and 147 mg/g, respectively, and are less than that for synthesized N-containing activated carbons, including PAnAC, PPyAC, and PnyAC adsorbents. 

## 4. Conclusions

In this work, nitrogen-containing activated carbons (PAnAC, PPyAC, and PnyAC) were synthesized from PAn and PPy precursors. The structural properties were elucidated via FTIR, SEM, BET, TGA, and CHN elemental analysis, supporting their potential as adsorbents. Thus, their adsorption performance in removing MO was assessed in terms of kinetic processes with variation in the adsorbate initial concentrations and adsorbent doses. The results revealed a better fit of experimental data to PSO, and their capacity was in the order PAnAC > PPyAC > PnyAC, supporting a dominant chemisorption process. The ad-sorption capacity was found to increase when MO initial concentration increased and slightly decreased as the adsorbent dose increased. The diffusion processes expressed by IPD and LFD models were found not to be the sole mechanism in the adsorption of MO onto ACs, while a complex mechanism is assumed. Hence, the high performance of such prepared nitrogen-doped graphene oxides makes the preparation route and the used precursors as promising candidates for the preparation of valuable materials from, e.g., nitrogen-containing plastic and textile wastes.

## Figures and Tables

**Table 1:** BET, CO_2_ adsorption capacity, and elemental analysis of the adsorbents, PAnAC, PPyAC, and PnyAC.

Material	BET Surface Area (m^2^/g)	Average Pore Width (nm)	CO_2_ Capacity (mg/g)	Elemental Composition (wt%)			
				C	H	N	O **
PAnAC *	1893	163.5	56.14	58.80	3.27	18.01	19.92
PPyAC	2157	158.9	48.41	73.46	0.57	6.95	19.02
PnyAC	1507	163.3	44.97	57.80	3.08	6.17	32.95

BET, Brunauer–Emmett–Teller; * taken from reference [17]; ** calculated values assuming total composition of 100%.

**Table 2:** Nonlinear PFO, PSO, and Elovich kinetic models.

Adsorbent	Conc. (mg/L)	*q*_e, exp_. (mg/g)	PFO			PSO			Elovich			Best Fit
			10^−3^*k*_1_ (min^−1^)	*q*_e, calc_. (mg/g)	*R* ^2^	10^−3^*k*_2_ (min^−1^)	*q*_e, calc_. (mg/g)	*R* ^2^	*α*	*β*	*R* ^2^	
PAnAC [17]	50	264.7	73.6	250.3	0.969	0.37	277.4	0.995	101.2	0.0214	0.985	2nd
	100	319.7	76.1	299.7	0.942	0.33	330.1	0.984	152.8	0.0178	0.992	Elovich
	200	405.0	84.4	368.9	0.957	0.30	405.9	0.992	223.1	0.0157	0.991	2nd
PPyAC	50	162.5	35.3	150.5	0.920	0.25	175.3	0.960	16.3	0.0272	0.986	Elovich
	100	190.3	28.9	185.7	0.986	0.14	225.8	0.994	11.4	0.0183	0.992	2nd
	200	203.6	12.9	188.4	0.933	0.85	205.5	0.963	245	0.0344	0.953	2nd
PnyAC	50	128.8	212.6	121.6	0.964	3.12	128.1	0.994	17,217	0.0954	0.991	2nd
	100	155.9	130.9	150.5	0.827	1.43	160.3	0.882	939.6	0.0556	0.869	2nd
	200	182.0	79.4	181.2	0.946	0.57	199.1	0.922	92.8	0.0312	0.846	1st

**Table 3:** Intraparticle diffusion (IPD) and liquid film diffusion (LFD) models.

Adsorbent	Conc. (mg/L)	*q*_e, exp_. (mg/g)	Diffusion					
			IPD			LFD		
			*k*_id_ (mg/g·min^0.5^)	C_id_ (mg/g)	*R* ^2^	*k*_fd_ (min^−1^)	C_fd_ (mg/g)	*R* ^2^
PAnAC	50	264.7	19.3	68.2	0.833	0.033	0.32	0.970
	100	319.7	22.7	86.4	0.840	0.038	0.22	0.948
	200	405.0	27.2	114.3	0.804	0.020	0.58	0.931
PPyAC	50	162.5	12.5	19.4	0.959	0.038	−0.42	0.728
	100	190.3	16.5	9.8	0.970	0.027	−0.03	0.956
	200	203.6	15.4	56.5	0.788	0.031	0.57	0.925
PnyAC	50	128.8	7.2	60.9	0.558	0.028	1.12	0.911
	100	155.9	9.9	63.6	0.661	0.033	1.10	0.868
	200	182.0	13.5	53.9	0.726	0.031	1.08	0.715

**Table 4:** Comparison of adsorption capacities of the investigated PAnAC, PPyAC, and PnyAC adsorbents with literature.

Adsorbent	Adsorbate	Capacity (*q*_e_; mg/g)	Condition: *C*_0_ (mg/L); Contact Time (*t*, min); Adsorbent Dosage (D, g/L); Temperature (*T*, °C); pH	Ref.
Merck; commercial activated carbon	Reactive violet 5	246	*C*_0_ = 1000; *t* = 150; D = 2.5; *T* = 25; pH = 2;	[52]
Cocoa shell-based acidified activated carbon		400		
Nitrogen-doped mesoporous carbons	Methyl orange	135	C_0_ = 200; *t* = 90; D = 1.0; *T* = 25;	[50]
Non-doped mesoporous carbons		120		
LOBA Chemie; commercial activated carbon		96	*C*_0_ = 80; *t* = 250; D = 0.75; *T* = 25; pH = 2.	[51]
Nitrogen-doped mesoporous carbon (NMC-3-800)		160	*C*_0_ = 300; *t* = 160; D = 1.0; *T* = 25;	[53]
Nitrogen-doped nonporous carbon (N-NC-800)		123	*C*_0_ = 50; *t* = 360; D = 0.4; *T* = 25; pH = 6.	[54]
Nitrogen-doped activated mesoporous carbon aerogel (NAMC)		354	*C*_0_ = 300; *t* = 180; D = 0.2; *T* = 25;	[55]
Polyaniline		111	*C*_0_ = 120; *t* = 20; D = 1.0; *T* = 25; pH = 7.	[56]
Polypyrrole		147	*C*_0_ = 150; *t* = 120; D = 1.0; *T* = 25; pH = 7.	[57]
Polyaniline-based activated carbon (PAnAC)		405	*C*_0_ = 200; *t* = 60; D = 0.16; *T* = 24; pH = 6.4;	[17]
Polypyrrole-based activated carbon (PPyAC)		204		This work
Poly(aniline-co-pyrrole)-based activated carbon (PnyAC)		182		

## Data Availability

The data that support the findings of this study are available from the corresponding author upon reasonable request.

## Supplementary Materials

The following are available online at https://www.mdpi.com/article/10.3390/polym15091983/s1, Figure S1: Kinetic adsorption plots of pseudo-first-order (PFO) models. Operating conditions: methyl orange (MO) initial concentration (C_0_) = 50, 100, and 200 ppm; MO volume = 250 mL; dosage = 40 mg; pH = 6.4; mixing speed = 150 rpm; temperature = 24 °C; contact time = 0–180 min. Figure S2: Kinetic adsorption plots of pseudo-second-order (PSO) models. Operating conditions: see Figure S1. Figure S3: Kinetic adsorption plots of Elovich models. Operating conditions: see Figure S1. Figure S4: The two-stage intraparticle diffusion model. Operating conditions: see Figure S1. Table S1: The linear and nonlinear equations for PFO, PSO, Elovich, IPD, and LFD kinetic models. Table S2: Thermogravimetric analysis (TGA) results for polymeric precursors, activated carbons before and after adsorption process. Table S3: Linear PFO, PSO, and Elovich and intraparticle kinetic models. Conditions: MO adsorbate concentration 50, 100, and 200 ppm; MO volume 250 mL; adsorbents PAnAC, PPyAC, and PnyAC dose 40 mg; pH of solutions 6.02–6.59; agitation speed 150 rpm; temperature 25 °C; contact time 0–180 min. Table S4: Adsorption capacities for the studied ad-sorbents from kinetic experiments performed using adsorbent doses of 20, 40, and 80 mg per 250 mL MO solution. Table S5: IPD two-stage results for MO adsorption onto activated carbons.
