# Supplementary material for: Adsorption Kinetics of Methyl Orange from Model Polluted Water onto N-Doped Activated Carbons Prepared from N-Containing Polymers
Source: Polymers (Basel). 2023 Apr 22;15(9):1983. doi: 10.3390/polym15091983 (PMC10180562; doi:10.3390/polym15091983)
Supplement: Supplementary file 1 [file polymers-15-01983-s001.zip › polymers-2322300-supplementary.pdf]

Article

Adsorption Kinetics of Methyl Orange from Model Polluted Water onto N-Doped Activated Carbons Prepared from N-Containing Polymers

Abdel-Basit Al-Odayni [1,*], Faisal S. Alsubaie [2], Naaser A. Y. Abdu [2], Haifa Masfeer Al-Kahtani [2], and Waseem Sharaf Saeed [1]

[1] Engineer Abdullah Bugshan Research Chair for Dental and Oral Rehabilitation, College of Dentistry, King Saud University, Riyadh 11545, Saudi Arabia
[2] Department of Chemistry, College of Science, King Saud University, Riyadh 11451, Saudi Arabia; 439106262@student.ksu.edu.sa (N.A.Y.A.)
* Correspondence: aalodayni@ksu.edu.sa

Supplementary Data:

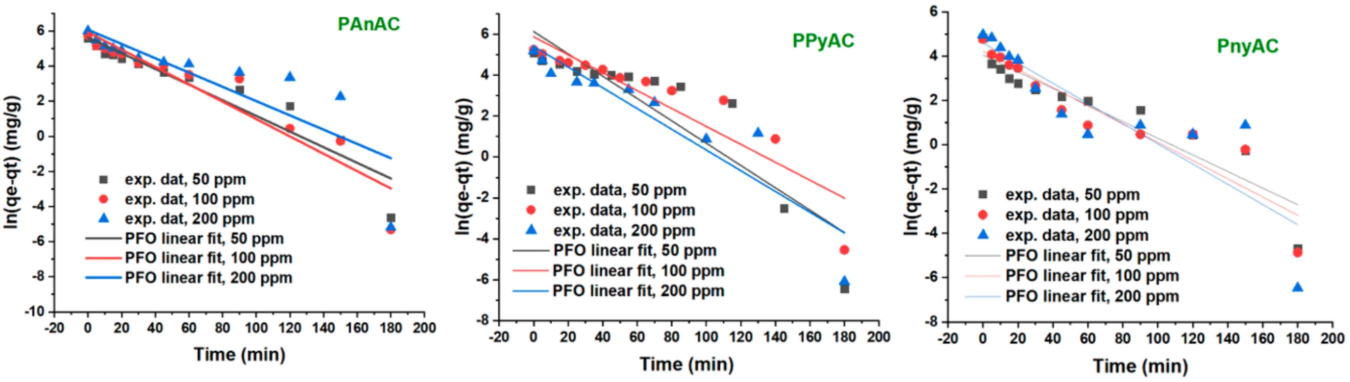

Figure S1. Kinetic adsorption plots of pseudo-first order (PFO) models. Operating conditions: methyl orange (MO) initial concentration (C_0) = 50, 100, and 200 ppm; MO volume = 250 mL; dosage = 40 mg; pH = 6.4; mixing speed = 150 rpm; temperature = 24 °C; contact time = 0–180 min.

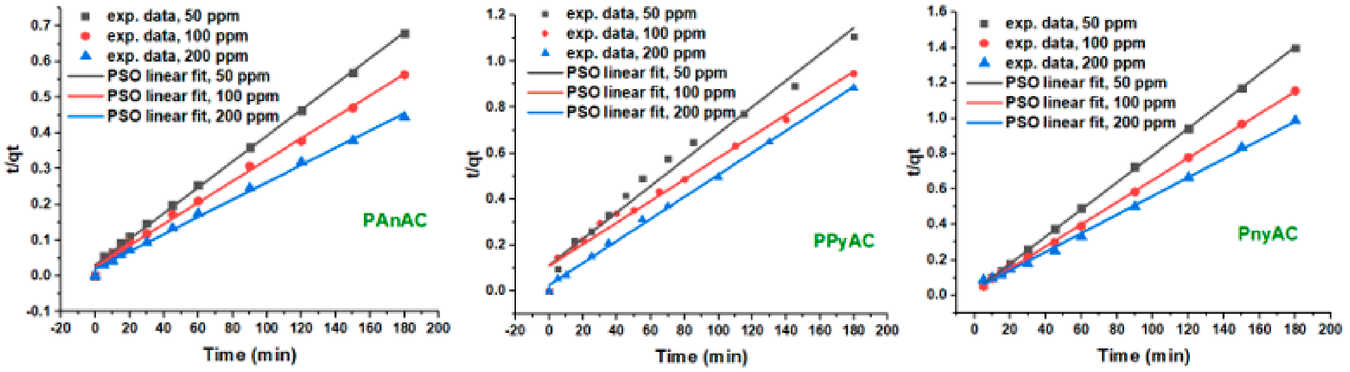

Figure S2. Kinetic adsorption plots of pseudo-second order (PSO) models. Operating conditions: see Figure S1.

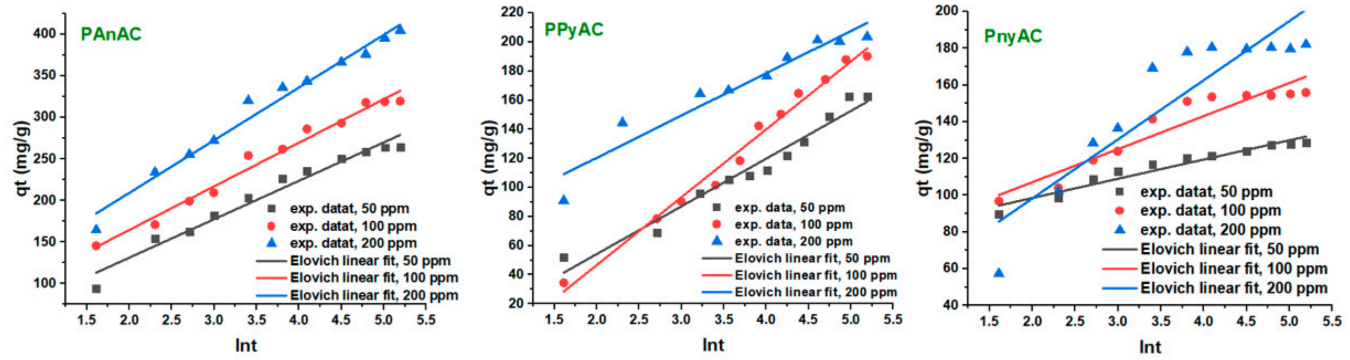

Figure S3. Kinetic adsorption plots of Elovich models. Operating conditions: see Figure S1.

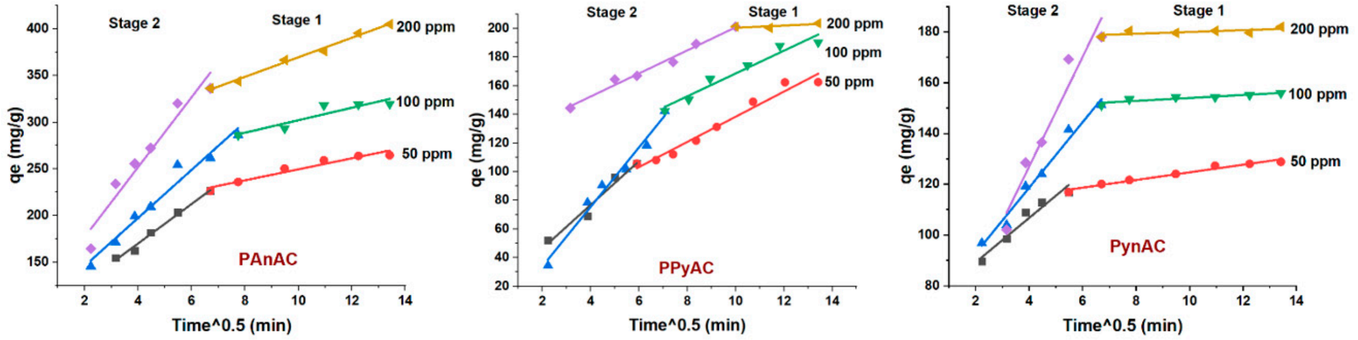

Figure S4. The two-stage intraparticle diffusion model. Operating conditions: see Figure S1.

Table S1. The linear and nonlinear equations for PFO, PSO, Elovich, IPD and LFD kinetic models

Kinetic models	Linear		Nonlinear		Constants	ref
	Equation	Plot	Equation	Plot		
PFO	$ln(q_e - q_t) = lnq_e - k_1 t$	$ln(q_e$-$q_t)$ vs. t	$q_t = q_e(1 - e^{-k_1 t})$	q_t vs. t	k_1 (min^{-1})	[1]
PSO	$\frac{t}{q_t} = \frac{1}{k_2 q_e t} + \frac{t}{q_t}$	t/q_t vs. t	$q_t = \frac{k_2 q_e^2 t}{1 + k_2 q_e t}$	q_t vs. t	k_2 (g/mg min); $h = k_2 q_e^2$ (mg/g min)	[2]
Elovich	$qt = \frac{1}{\beta} ln(\alpha\beta) + \frac{1}{\beta} lnt$	q_t vs. lnt	$qt = \frac{1}{\beta} \ln(1 + \alpha\beta t)$	q_t vs. t	α (mg/g min); β (g/mg)	[3]
IPD	$q_t = k_{id} t^{0.5} + C$	q_t vs. $t^{0.5}$	-	-	k_{id} (mg/g $min^{0.5}$); C (mg/g)	[4]
LFD	$-\ln(1 - F) = k_{fd} t + C$, $F = \frac{q_t}{q_e}$	$-\ln(1 - F)$ vs. t	-	-	k_{fd} (min^{-1}); C (mg/g)	[5]

Table S2. Thermogravimetric analysis (TGA) results for polymeric precursors, activated carbons before and after adsorption process.

Steps	Property	PAn	PPy	Pny	PAnAC	PPyAC	PnyAC	PAnAC-MO	PPyAC-MO	PnyAC-MO
Step 1	Temp. (°C)	25–153	25–153	25–153	25–153	25–153	25–153	25–208	25–208	25–208
	Mass Loss (%)	7.4	6.0	6.7	10.9	12.1	13.5	11.2	11.8	9.8
	DTG (°C)	95	85	105	86	81	77	98	103	92
	Assig.	Adsorbed volatiles								
Step 2	Temp. (°C)	154–255	154–255	154–255	154–326	154–326	154–417	209–474	209–474	209–474
	Mass Loss (%)	8.2	6.2	7.8	6.0	7.1	7.1	14.3	15.3	9.6
	DTG (°C)	221	230	229	248	268	366	381	378	370
	Assig.	Weak parts and sensitive functional groups								
Step 3	Temp. (°C)	256–419	256–419	256–419	327–417	327–785	-	-	-	-
	Mass Loss (%)	14.7	17.2	15.5	2.5	23	-	-	-	-
	DTG (°C)	300	285	289	377	531	-	-	-	-
	Assig.	Oxidative process				Backbone decomp.	-	-	-	-
Step 4	Temp. (°C)	420–686	420–850	420–686	418–785	-	-	-	-	-
	Mass Loss (%)	18.9	20.5	17.8	13.8	-	-	-	-	-
	DTG (°C)	559	-	537	580	-	-	-	-	-
	Assig.	Carbonaceous step (C. step)				-	-	-	-	-
Step 5	Temp. (°C)	687–950	-	687–950	-	-	-	-	-	-
	Mass Loss (%)	7.4	-	7.7	-	-	-	-	-	-
	DTG (°C)	771	-	769	-	-	-	-	-	-
	Assig.	C. step	-	C. step	-	-	-	-	-	-
Residue at 950 °C		43.4	50.1	44.5	64.5	54.7	70.6	62.3	57.7	73.0

Table S3. Linear PFO, PSO, and Elovich and intraparticle kinetic models. Conditions: MO adsorbate concentration 50, 100, and 200 ppm; MO volume 250 mL; adsorbents PAnAC, PPyAC, and PnyAC dose 40 mg; pH of solutions 6.02–6.59; agitation speed 150 rpm; temperature 25 °C; contact time 0–180 min.

Adsorbent	Conc. (mg/L)	$q_{e, exp.}$ (mg/g)	PFO linear			PSO linear			Elovich linear			Best fit
			$10^{-3}k_1$ (min^{-1})	$q_{e, calc.}$ (mg/g)	R^2	$10^{-3}k_2$ (min^{-1})	$q_{e, calc.}$ (mg/g)	R^2	α	β	R^2	
PAnAC	50	264.7	44.6	276.5	0.894	0.43	276.2	0.998	106.7	0.0216	0.968	2nd
	100	319.7	49.5	376.5	0.896	0.35	333.3	0.997	163.2	0.0190	0.980	2nd
	200	405.0	40.7	433.7	0.706	0.29	413.2	0.997	232.5	0.0158	0.978	2nd
PPyAC	50	162.5	54.6	461.4	0.807	0.29	174.5	0.974	23.2	0.0305	0.962	2nd
	100	190.3	43.8	355.4	0.838	0.20	213.2	0.978	17.2	0.0215	0.988	2nd
	200	203.6	50.7	223.5	0.857	0.83	208.8	0.998	248.6	0.0344	0.924	2nd
PnyAC	50	128.8	37.5	57.2	0.868	2.24	130.7	0.999	17216	0.0955	0.942	2nd
	100	155.9	41.2	68.4	0.884	1.51	160.0	0.999	941.4	0.0556	0.898	2nd
	200	182.0	45.6	99.5	0.780	0.78	190.1	0.998	92.4	0.0310	0.829	2nd

Table S4. Adsorption capacities for the studied adsorbents from kinetic experiments performed using adsorbent doses of 20, 40, and 80 mg per 250 mL MO solution.

Adsorbent	Dose (mg)		
	20 (mg)	40 (mg)	80 (mg)
PAnAC; $q_{e,exp.}$ (mg/g)	318.09	319.73	287.73
PPyAC; $q_{e,exp.}$ (mg/g)	273.34	190.30	247.76
PnyAC; $q_{e,exp.}$ (mg/g)	163.04	155.93	161.04

Table S5. IPD two-stage results for MO adsorption onto activated carbons

Adsorbent	Conc. (mg/L)	$q_{e, exp.}$ (mg/g)	IPD model					
			Stage 1			Stage 2		
			k_{id1} (mg/g·min$^{0.5}$)	C_{id1} (mg/g)	$(R_1)^2$	k_{id2} (mg/g·min$^{0.5}$)	C_{id2} (mg/g)	$(R_2)^2$
PAnAC	50	264.7	21.16	85.35	0.990	5.91	190.40	0.936
	100	319.7	25.74	94.39	0.966	6.69	235.29	0.854
	200	405.0	37.46	101.94	0.9939	10.49	264.75	0.992
PPyAC	50	162.5	15.28	15.67	0.69	9.13	46.64	0.955
	100	190.3	20.78	-7.77	0.980	7.96	88.93	0.951
	200	203.6	8.06	120.24	0.969	0.76	192.88	0.621
PnyAC	50	128.8	71.71	8.75	0.948	109.60	1.51	0.970
	100	155.9	66.97	12.91	0.981	148.16	0.58	0.846
	200	182.0	40.40	21.62	0.941	176.39	0.36	0.511

References

1. Lagegren, S.; Svenska, B. Zur theorie der sogenannten adsorption geloester stoffe. *Vaternskapsakad Handlingar* **1898**, *24*, 1-39.
2. Ho, Y.-S.; McKay, G. Pseudo-second order model for sorption processes. *Process biochemistry* **1999**, *34*, 451-465.
3. López-Luna, J.; Ramírez-Montes, L.E.; Martinez-Vargas, S.; Martínez, A.I.; Mijangos-Ricardez, O.F.; González-Chávez, M.d.C.A.; Carrillo-González, R.; Solís-Domínguez, F.A.; Cuevas-Díaz, M.d.C.; Vázquez-Hipólito, V. Linear and nonlinear kinetic and isotherm adsorption models for arsenic removal by manganese ferrite nanoparticles. *SN Applied Sciences* **2019**, *1*, 1-19.
4. Weber Jr, W.J.; Morris, J.C. Kinetics of adsorption on carbon from solution. *Journal of the sanitary engineering division* **1963**, *89*, 31-59.
5. Ali, I.; Alothman, Z.A.; Alwarthan, A. Uptake of propranolol on ionic liquid iron nanocomposite adsorbent: kinetic, thermodynamics and mechanism of adsorption. *Journal of Molecular Liquids* **2017**, *236*, 205-213.
